# Neuroendocrine Differentiation in Metastatic Conventional Prostate Cancer Is Significantly Increased in Lymph Node Metastases Compared to the Primary Tumors

**DOI:** 10.3390/ijms18081640

**Published:** 2017-07-28

**Authors:** Vera Genitsch, Inti Zlobec, Roland Seiler, George N. Thalmann, Achim Fleischmann

**Affiliations:** 1 Institute of Pathology, University of Bern, Bern 3008, Switzerland; vera.genitsch@pathology.unibe.ch (V.G.); inti.zlobec@pathology.unibe.ch (I.Z.); 2 Department of Urology, University of Bern, Bern 3010, Switzerland; roland.seiler@insel.ch (R.S.); george.thalmann@insel.ch (G.N.T.)

**Keywords:** prostate cancer, lymph node metastases, neuroendocrine, chromogranin A, prognosis

## Abstract

Neuroendocrine serum markers released from prostate cancers have been proposed for monitoring disease and predicting survival. However, neuroendocrine differentiation (NED) in various tissue compartments of metastatic prostate cancer is poorly described and its correlation with specific tumor features is unclear. NED was determined by Chromogranin A expression on immunostains from a tissue microarray of 119 nodal positive, hormone treatment-naïve prostate cancer patients who underwent radical prostatectomy and extended lymphadenectomy. NED in the primary cancer and in the metastases was correlated with tumor features and survival. The mean percentage of NED cells increased significantly (*p* < 0.001) from normal prostate glands (0.4%), to primary prostate cancer (1.0%) and nodal metastases (2.6%). In primary tumors and nodal metastases, tumor areas with higher Gleason patterns tended to display a higher NED, although no significance was reached. The same was observed in patients with a larger primary tumor volume and higher total size and number of metastases. NED neither in the primary tumors nor in the metastases predicted outcome significantly. Our data suggest that (a) increasing levels of neuroendocrine serum markers in the course of prostate cancer might primarily derive from a poorly differentiated metastatic tumor component; and (b) NED in conventional hormone-naïve prostate cancers is not significantly linked to adverse tumor features.

## 1. Introduction

The current World Health Organization (WHO) classification of prostate neoplasms with neuroendocrine (NE) differentiation (NED) comprises of: (1) adenocarcinomas with NED; (2) well-differentiated NE tumors (carcinoid); (3) small-cell NE carcinomas; and (4) large cell NE carcinomas [1]. While the last three entities are exceedingly rare, the first occurs frequently. In 10–100% of the conventional adenocarcinomas, NED can be demonstrated immunohistochemically in the form of scattered NE cancer cells, depending on the number of slides evaluated and the number of antibodies used [1].

NE cells in prostate cancer most likely emerge from the secretory prostate cancer cells by trans-differentiation [2,3,4]. Each NE cell may store a single, or a mix of neuropeptides in cytoplasmic granules, including Chromogranin A, the most frequently detected and most intensely studied NE product in prostate tissue [5], serotonin, somatostatin and bombesin [6]. The exact biological function of neuropeptides in prostate cancer is largely unknown; however, data indicate that they may stimulate growth, differentiation and secretory processes [5,7]. While small and large cell NE carcinomas are particularly aggressive [8], the prognostic significance of NE cells in conventional adenocarcinomas of the prostate is still controversial [4,9,10]. Importantly, neuropeptides released from the NE prostate cancer cells may appear in the circulation [6]. These serum markers have recently attracted considerable attention for their ability to monitor disease [6,11] and predict survival [12,13]. NE serum markers have been suggested as beneficial surrogates for tumor burden [6] and mirror prostate cancer progression when raising. In line with this, serum levels of Chromogranin A are significantly higher in metastatic compared to non-metastatic prostate cancers [14]. However, despite this interest in NE serum markers, little is known about the distribution of their source, which are the NE tumor cells, in the various growth patterns and in the metastases of prostate cancer. In this study, we more accurately describe the extent of NED in the different tissue compartments of metastasizing prostate cancer, and determine its correlations with different tumor features and survival.

## 2. Results

### 2.1. Patient Characteristics and Expression of Chromogranin A in Benign Prostate, Primary Tumors and Lymph Node Metastases Considering the Gleason Patterns

**Figure 1:**
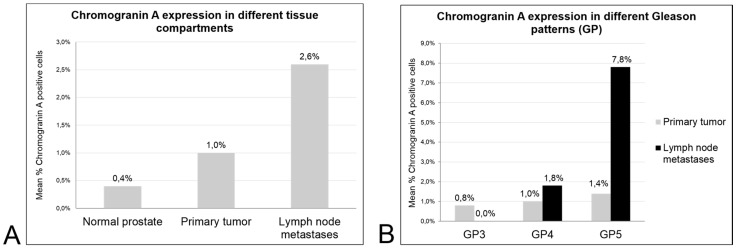
Mean density of Chromogranin A positive cells is significantly different between normal prostate glands, primary prostate cancer and matched lymph node metastases ((**A**) *p* < 0.001). The difference between the Gleason patterns is not significant ((**B**) *p* > 0.05).

The patient, prostatectomy and lymphadenectomy characteristics are specified in Table 1. A higher proportion of 92% of primary tumors displayed any positivity for Chromogranin A compared to lymph node metastases with a positive expression in 77%. When the density of NE cancer cells was recorded, a progressive and significant increase in expression from non-neoplastic prostate glands (0.4% mean of Chromogranin A positive cells) to primary tumors (1.0%) and lymph node metastases (2.6%; *p* < 0.001) was noted for Chromogranin A (Figure 1A).

A tendency for higher Chromogranin A expression in less-differentiated tumor areas (reflected by a higher Gleason pattern (GP)) was observed in the primary tumors (GP3: 0.8% mean of Chromogranin A positive cells; GP4: 1.0%; GP5: 1.4%; *p* > 0.05) and in the nodal metastases (GP3: 0.0%; GP4: 1.8%; GP5: 7.8%; P = NE), but no statistical significance was reached (Figure 1B).

### 2.2. Correlations of Chromogranin A Expression in Primary Tumors and Lymph Node Metastases with Clinico-Pathological Tumor Characteristics and Survival

**Figure 2:**
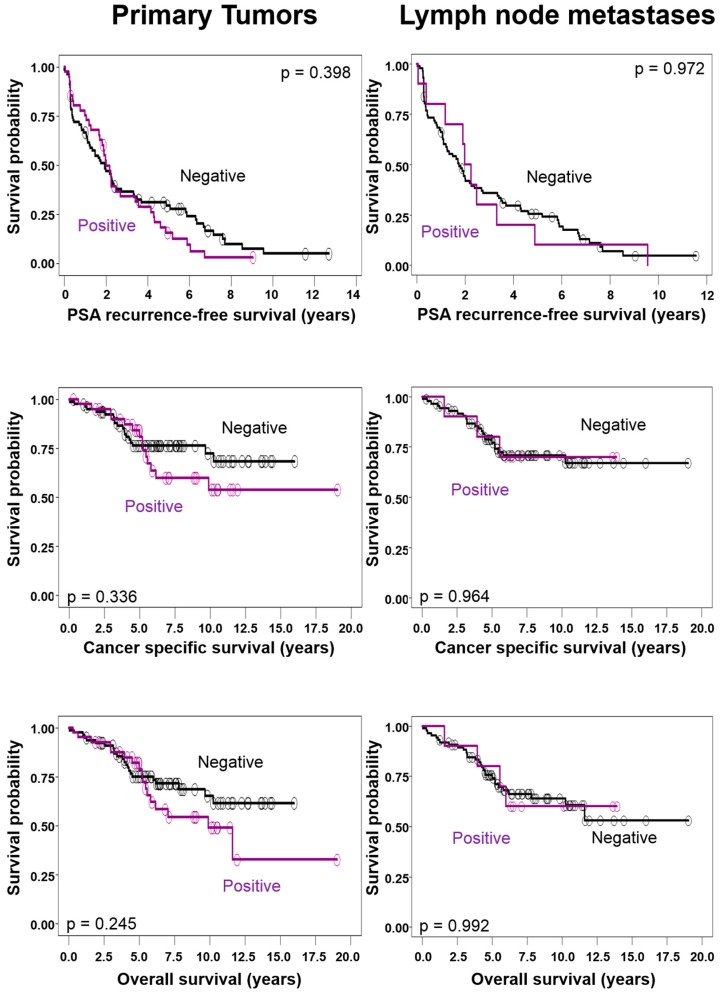
Chromogranin A expression in primary tumors and metastases is not significantly correlated with outcome.

Primary tumors with Chromogranin A expression were larger (mean 21.5 ± 24.9 cm^3^ versus 18.0 ± 15.4 cm^3^; *p* = 0.821) and the tumor burden of a Chromogranin A positive metastasizing component was higher for mean total size and number of metastases (36.4 ± 49.4 mm versus 19.4 ± 31.7 mm; *p* = 0.458 and 5.3 ± 6.9 versus 3.3 ± 3.4; *p* = 0.279) (Table 2); however, these differences were not statistically significant. Chromogranin A expression in primary tumors or lymph node metastases was not associated with categorical tumor characteristics as stage of the primary tumor. In univariate analysis, Chromogranin A expression in primary tumors or lymph node metastases did not significantly predict biochemical recurrence-free, cancer-specific, or overall survival (Figure 2). Only the total size of metastases independently predicted all three endpoints in a multivariate analysis (Table 3).

## 3. Discussion

Only a few studies on prostate cancers have evaluated NED in metastatic tissues from lymph nodes and various other organs with immunohistochemistry [15,16,17,18,19,20]. Reported incidences for bone metastases were 19% [18] and 52% [16], those for lymph node metastases 12% [19], 37.5% [17] and 46% [16]. A wide range in the extent of NED in metastases was also noted in an autopsy series by Roudier et al. [20], specifically between patients, and also between different metastases of a single patient. In our series, NED in lymph node metastases was present in 77% of the patients. The metastases had a lower prevalence for NED positivity compared to the primary tumors, which showed NE differentiation in 92%. This decrease was consistent with the only two series on NED in surgically treated nodal positive prostate cancer reported by Bostwick et al. [17] and Quek et al. [19]. However, when considering not only the presence or absence of NED, but also the density of positive cells in primary tumors and metastases, we noticed a significant increase in NED in metastases when compared to primary tumors. Furthermore, NED increased in higher Gleason patterns in the primary tumors, and was even more striking in the metastases where tumor growths of Gleason pattern 5 showed the highest levels of NED among all evaluated cancer components. Our findings were consistent with reports on a positive correlation of the extent of NED and the Gleason score in primary tumors [21,22]. Together with the previously described correlation of Chromogranin A expression by the tumor tissue with its serum level [23], our data might suggest that elevated NE serum markers in metastatic prostate cancer [14] may primarily reflect the metastatic, frequently poorly differentiated tumor burden [24,25,26].

The presence of NED in our prostate cancer patients showed a tendency for association with adverse tumor characteristics. Patients with detected NED in primary tumors had larger tumors, and those with NED present in metastases had a greater nodal tumor burden, indicated by more metastases and greater total diameter of metastases when compared with patients without NED. Consistent with our data, Quek et al. [19] reported the association of high NED with an advanced tumor stage. Furthermore, NED in the primary tumors of our patients translated into long-term survival. After five years, the curves for disease-specific and overall survival segregate clearly indicated a poorer outcome for patients with NED when compared to those without NED. However, this was not significant, most likely due to the size of our cohort. Contrarily, survival curves based on NED in lymph node metastases intersected repeatedly. Only two other studies have evaluated NED in nodal positive prostate cancer patients treated by radical prostatectomy and bilateral lymphadenectomy. NED detected by Chromogranin A was not a risk factor in the study by Bostwick et al. [17], neither in the primary tumors nor in the metastases, whereas Queck et al. [19] reported significantly poorer median recurrence-free and overall survival for patients with high NED in the primary tumor and metastases, respectively, when compared to patients with low NED. However, survival curves were not presented in the latter study and other outcome measures were not significantly different.

Previous studies on NED in prostate cancer tissues assessed expression on large sections (for comprehensive review of the literature see Table 3 in Bostwick et al. [17]) and cancers were categorized as negative (absence of NE cells), or positive (presence of NE cells). While it was generally noticed that NED in prostate cancer is a very focal, dispersed phenomenon, reported incidences for NED varied between 24 and 98.5% [17]. This wide range was attributed to differences between the cohorts, sample types, types and extent of fixation, the antibodies used in determining the presence of malignant NE cells, variance in interpretation and, most importantly, a sampling error related to the focal and unequal distribution of NE cells in most tumors [27]. It is evident that the amount of tumor tissue evaluated may impact on reported prevalence in cases of only focally expressed biomarkers like NED. We determined NED in primary tumors and metastases by tissue microarray (TMA). This technology has also been considered to be useful for these focally expressed biomarkers in prostate cancer by a study comparing the expression of NE markers on whole tissue sections to a TMA [28]. Investigating these focally expressed biomarkers on large sections may have also been problematic as tissue slides from primary prostate cancer generally contain much greater amounts of tumor tissue than the usually scarce metastatic tissue that makes the comparison of incidences difficult. However, the use of a TMA certainly remains a limiting factor in our study. Finally, for a delicate biomarker like NED in prostate cancer, the size of the cohort may play a major role in detecting significant correlations between tumor features and survival. Our cohort was comparably large for surgically treated nodal positive prostate cancer and therefore allowed detection of a significant increase in NED in nodal metastases and trends between biomarker expression levels, tumor features and survival. However, it may have been too small to demonstrate these trends as significant.

## 4. Materials and Methods

### 4.1. Patients

In total, 119 consecutive prostate cancer patients without demonstrable metastases (physical and radiological examination), but with nodal metastases upon histological investigation of the lymphadenectomy specimens were studied. All patients had undergone standardized surgery at the Department of Urology, University of Bern between 1989 and 2006 with bilateral extended pelvic lymphadenectomy and radical prostatectomy as a single procedure. Follow-up was performed prospectively. Neoadjuvant therapy was not implemented and no adjuvant treatment, especially androgen deprivation, was suggested before symptomatic disease progression.

### 4.2. Surgical Technique of Lymphadenectomy

A bilateral pelvic lymphadenectomy was performed in all patients as previously described [29]. Summarized, lymph nodes were dissected along the external iliac vein down to the deep circumflex iliac vein and femoral canal, up to the bifurcation of the common iliac artery and the obturator fossa. Thereafter, the lymphatic tissue along the medial and lateral aspect of the internal iliac artery and vein was excised. Three tissue samples from each side were submitted separately for pathological examination. Frozen sections were not carried out.

### 4.3. Pathology

All specimens were processed at the Institute of Pathology, University of Bern [24,30]. The prostatectomies were completely embedded as described in references [24,30]. The following microscopic tumor characteristics were noted: type, Gleason score [31], tertiary Gleason pattern, tumor stage, and the percentage of prostate tissue area on the sections occupied by the tumor. NE tumors/carcinomas of the prostate were excluded. Tumor volume was estimated by multiplying the percentage of the specimen involved by cancer by the prostate volume.

The fatty tissue of lymphadenectomy specimens was dissolved in aceton after formalin fixation and all lymph nodes were entirely embedded. One section per paraffin block was stained with hematoxylin and eosin. The length and width of the metastatic deposits were measured. A Gleason score (primary and secondary pattern) and a tertiary Gleason pattern (if present), were determined based on the entire metastatic tissue.

All Gleason patterns present in the primary tumors and lymph node metastases were accurately marked for subsequent TMA construction. Staging was completed according to the 8th edition of the International Union Against Cancer (UICC) TNM Classification [32].

### 4.4. Tissue Microarray

For TMA construction, one 0.6 mm tissue core of benign prostatic tissue (peripheral zone) and every Gleason pattern present in primary tumors and matched lymph node metastases was retrieved from the paraffin blocks. The TMA contains overall 403 prostate tissue samples, 119 normal prostate tissues and 284 primary cancers (mean per patient, 3.3; range, 2–4; including 101, 112 and 71 samples from Gleason patterns 3, 4 and 5, respectively) and 167 lymph node metastases (mean per patient, 1.4; range, 1–3; including 35, 103 and 29 samples from Gleason patterns 3, 4 and 5, respectively). In the vast majority of primary tumors, all Gleason patterns sampled were present in the index tumor. Additional tissue from separate tumor foci was included only rarely, when a Gleason pattern not present in the index tumor was detected here. Although sampling from the primary tumor was more extensive, the relative tumor amount in the TMA was larger from the metastases due to their smaller volume.

### 4.5. Immunohistochemistry

**Figure 3:**
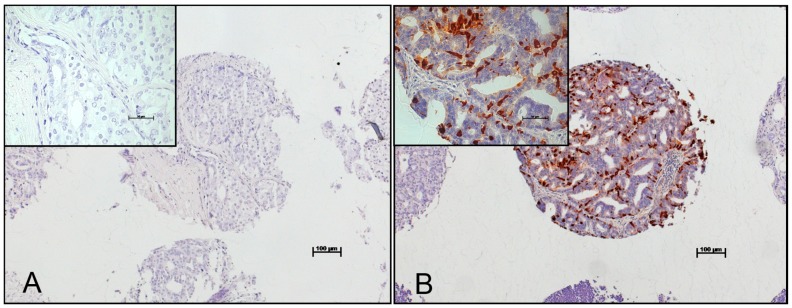
No Chromogranin A expression in (**A**) primary prostate cancer; and (**B**) high Chromogranin A expression in a lymph node metastasis.

Freshly cut TMA sections were pre-treated by steam with target retrieval solution, pH 9 (Dako, Glostrup, Denmark). For Chromogranin A detection, a monoclonal mouse antibody cocktail (clone LK2H10 + PHE5; Bicarta; Hamburg, Germany) was used at 1:500 antibody dilution. Bound primary antibodies were detected using the Envision Plus system (Dako, Glostrup, Denmark). Chromogranin A was expressed in the cytoplasm of the prostate cancer cells (Figure 3). The percentage of positive neoplastic cells was determined for every tissue sample.

### 4.6. Statistical Analysis

Chromogranin A expression in normal prostate, primary tumors and lymph node metastases was evaluated using the Wilcoxon Signed Rank test and the Friedman test for differences between Gleason pattern 3, 4 and 5 within primary carcinomas and nodal metastases. Chromogranin A expression was compared with normally distributed quantitative and categorical tumor attributes using Wilcoxon Signed Rank test and χ-Square test, respectively. Suitable cut-off values for positive (more than 0 positive cells) and negative (0 positive cells) Chromogranin A expression in primary tumors and lymph node metastases were defined using Receiver-operating characteristic curves [33]. Outcome was analyzed for Prostate-Specific Antigen (PSA) recurrence-free, cancer-specific and overall survival defined as the intervals from surgery to the date of biochemical recurrence (PSA failure defined as values >0.2 ng/mL), death from prostate carcinoma, and death from any cause, respectively. Patients without event for the respective endpoints were censored at the date of last follow-up. The above time-to-events were performed using log-rank test; *p* values < 0.05 were regarded as significant. The Cox proportional hazards model was used to identify independent prognostic factors for all three endpoints. Statistical analysis was made using SAS 9.2 (The SAS Institute, Cary, NC, USA).

## 5. Conclusions

Our data suggest that, firstly, increasing serum levels of neuroendocrine serum markers in prostate cancer primarily mirror growth of a poorly differentiated metastatic tumor component and, secondly, NED in early metastasizing, hormone-naïve prostate cancer is only weakly linked to adverse tumor features.

## Figures and Tables

**Table 1:** Characteristics of 119 nodal positive prostate cancer patients.

**Patient Data (*n* = 119)**	
Age (median, range) at surgery (years)	65 (45–75)
Follow-up (median, range) (years)	5.9 (0.1–15.2)
Patients with biochemical failure at last follow-up (*n*)	103
Patients dead of disease at last follow-up (*n*)	33
Patients dead at last follow-up (*n*)	40
**Prostatectomy Data**	
pT2 (*n*)	14
pT3a (*n*)	55
pT3b (*n*)	50
Prostate cancer volume (median, range) (cm^3^)	12.6 (0.66–127)
Gleason score 6 (*n*)	12
Gleason score 7 (*n*)	63
Gleason score 8 (*n*)	21
Gleason score 9 (*n*)	23
**Lymphadenectomy Data**	
Evaluated nodes per patient (median, range) (*n*)	22 (9–68)
Positive nodes per patient (median, range) (*n*)	2 (1–24)

**Table 2:** Tumor features according to Chromogranin A expression.

CgA Expression	Parameters of the Primary Tumor (Mean ± SD)				Parameters of Nodal Metastases (Mean ± SD)			
	Age	*p*	Tumor volume (cm^3^)	*p*	Total size (mm)	*p*	Total number	*p*
**Primary Tumor**								
CgA negative	64.4 ± 6.1	0.978	18.0 ± 15.4	0.821	19.6 ± 34.8	0.989	3.3 ± 3.8	0.813
CgA positive	64.3 ± 5.8		21.5 ± 24.9		17.2 ± 24.4		3.0 ± 3.3	
**Nodal Metastases**								
CgA negative	64.3 ± 5.9	0.027	19.1 ± 19.5	0.819	19.4 ± 31.7	0.458	3.3 ± 3.4	0.279
CgA positive	59.3 ± 6.3		18.9 ± 13.7		36.4 ± 49.4		5.3 ± 6.9	

**Table 3:** Multivariate analyses for the prognostic impact of Chromogranin A (CgA) expression in primary prostate cancer (upper half) and in lymph node metastases (lower half), after adjustment for total size of metastases and Gleason score of primary tumor: Only nodal tumor burden predicts survival independently. HR, hazard ratio; and CI, confidence interval.

Parameter	Cut-Off	Overall Survival		Disease-Specific Survival		Recurrence-Free Survival	
		HR (95% CI)	*p*	HR (95% CI)	*p*	HR (95% CI)	*p*
**CgA in Primary Tumor**	Positive	1.0	0.132	1.0	0.241	1.0	0.66
	Negative	1.65 (0.9–3.1)		1.54 (0.8–3.1)		1.1 (0.7–1.7)	
Metastases size	<7.5 mm	1.0	**<0.001**	1.0	**0.002**	1.0	**0.036**
	≥7.5 mm	4.34 (2.0–9.6)		4.12 (1.7–10.0)		1.58 (1.1–2.4)	
Gleason score	6 to 8	1.0	0.571	1.0	0.375	1.0	0.074
	9 to 10	1.23 (0.6–2.5)		1.41 (0.7–3.0)		1.57 (1.0–2.6)	
**CgA in Nodal Metastases**	Positive	1.0	0.571	1.0	0.5	1.0	0.327
	Negative	0.73 (0.2–2.2)		0.65 (0.2–2.8)		0.69 (0.3–1.4)	
Metastases size	<7.5 mm	1.0	**<0.001**	1.0	**0.003**	1.0	0.063
	≥7.5 mm	5.3 (2.0–14.1)		6.44 (1.9–22.1)		1.58 (1.0–2.5)	
Gleason score	6 to 8	1.0	0.365	1.0	1.88	1.0	0.082
	9 to 10	1.43 (0.7–3.1)		1.75 (0.8–4.0)		1.62 (0.9–2.8)	

## Abbreviations

NED	Neuroendocrine Differentiation
NE	Neuroendocrine
GP	Gleason Pattern
CgA	Chromogranin A
HR	Hazard Ratio
CI	Confidence Interval
TMA	Tissue Microarray
PSA	Prostate-Specific Antigen
